# The Epidemiological Characteristics and Prognostic Factors of Low-Grade Brainstem Glioma: A Real-World Study of Pediatric and Adult Patients

**DOI:** 10.3389/fonc.2020.00391

**Published:** 2020-04-09

**Authors:** Zhuoyi Liu, Songshan Feng, Jing Li, Hui Cao, Jun Huang, Fan Fan, Li Cheng, Zhixiong Liu, Quan Cheng

**Affiliations:** ^1^Department of Neurosurgery, Xiangya Hospital, Center South University, Changsha, China; ^2^National Clinical Research Center for Geriatric Disorders, Xiangya Hospital, Central South University, Changsha, China; ^3^Department of Rehabilitation, Second Xiangya Hospital, Central South University, Changsha, China; ^4^Department of Psychiatry, The Second People's Hospital of Hunan Province, The Hospital of Hunan University of Chinese Medicine, Changsha, China; ^5^Center for Medical Genetics and Hunan Provincial Key Laboratory of Medical Genetics, School of Life Sciences, Central South University, Changsha, China; ^6^Department of Emergency, Fengyang County Hospital of Traditional Chinese Medicine, Fengyang, China; ^7^Department of Clinical Pharmacology, Xiangya Hospital, Central South University, Changsha, China

**Keywords:** low-grade brainstem glioma, SEER, cancer-specific survival, overall survival, nomogram, real-world study

## Abstract

**Purpose:** Our current understanding of low-grade brainstem glioma (LGBSG) is still limited. This study aimed to conduct a large-scale population-based real-world study to understand the epidemiological characteristics of LGBSG and determine the predictive factors of cancer-specific survival (CSS) and overall survival (OS) of LGBSG patients.

**Patients and Methods:** We used Surveillance Epidemiology and End Results database to conduct this study of patients with histologically confirmed LGBSG. Patient demographics, tumor characteristics, and treatment options were compared between pediatric and adult patients. Univariate and multivariate analyses were employed to determine prognostic factors of CSS and OS. Kaplan–Meier curve and decision tree were used to confirm the prognostic factors. All variables were further identified by L1-penalized (Lasso) regression and then a nomogram was established to predict the 5- and 8-year CSS and OS rate. The precision of the nomogram was evaluated by calibration plots, Harrell's concordance index, and time-dependent receiver operating characteristic curve. The clinical use of nomogram was estimated by decision curve analysis.

**Results:** A cohort of 305 patients with LGBSG, including 165 pediatric and 140 adult patients, was analyzed. Adult and pediatric patients showed different patterns concerning tumor size, tumor extension, adjuvant therapy, and survival rate. Univariate analysis revealed that pediatric group, gross total resection (GTR), World Health Organization grade II, radiotherapy, extension to ventricular system, and diffuse astrocytic and oligodendroglial tumor (DAOT) were significantly associated with CSS. Multivariate analysis showed that pediatric group, metastasis, ventricular system involvement, and DAOT were independently associated with CSS. The prognostic factors were further confirmed by Kaplan–Meier curve and decision tree. Kaplan–Meier curve also showed that adjuvant therapy added no benefits in patients with GTR and non-GTR. In addition, the nomogram was developed and the C-index of internal validation for CSS was 0.87 (95% CI, 0.78–0.96).

**Conclusion:** This study shows that pediatric and adult patients have different tumor characteristics, treatment options, and survival rate. Pediatric group, DAOT, ventricular system involvement, and metastasis were identified as independent prognostic factors for CSS by multivariate analysis. Adjuvant therapy showed no benefits on CSS in patients with GTR and non-GTR. The nomogram was discriminative and clinically useful.

## Introduction

Brainstem glioma (BSG) encompasses a heterogeneous group of tumors, which are classified according to epidemiological, imaging [magnetic resonance imaging (MRI)], and pathological characteristics. Epidemiologically, BSG accounts for 4.3% of all gliomas as recorded in the most recent Central Brain Tumor Registry of the United States (CBTRUS) report (1). Notably, BSG constitutes ~15% of pediatric brain tumors and <2% of adult gliomas (1, 2). Based on MRI characteristics and surgical experience, Choux et al. (3) classified BSG into types of diffuse, intrinsic focal, extrinsic focal, and cervicomedullary, and this remains to be most recent and widely accepted categorization system of BSG. Diffuse brainstem pontine glioma (DIPG) is associated with dismal prognosis in both pediatric and adult patients, being highly infiltrative and less amenable to surgery (4, 5). Pathologically, pediatric and adult patients with high-grade BSG (HGBSG), including World Health Organization (WHO) grades III and IV BSG, have worse clinical outcomes (6, 7). Over the last two decades, we have gained deep understanding on pediatric DIPG and HGBSG in terms of biological characteristics, prognostic factors, and treatment strategies (8–11). However, little is known about low-grade BSG (LGBSG) especially its presentation in adult patients. Only few single-center retrospective studies with small population concerning pediatric LGBSG were available. And no study focusing on adult LGBSG has been published to date. Regarding its treatment modalities, surgical resection has improved with advancing imaging and neurosurgery techniques (12–14). At the same time, efforts were devoted to investigate the adjuvant therapy including radiotherapy (RT) and chemotherapy (CT) for LGBSG patients (15–19). However, there is no consensus on the benefits of surgical resection and adjuvant therapy for LGBSG.

This population-based real-world study was conducted to address this challenge. A search was performed on the SEER (Surveillance Epidemiology and End Results) database, which identified 165 pediatric and 140 adult patients with histologically confirmed LGBSG from 2004 to 2015. The major purpose of this study was to determine the prognostic factors influencing cancer-specific survival (CSS) and overall survival (OS), which could help to optimize the management of patients with LGBSG.

## Methods

### Study Population

**Figure 1:**
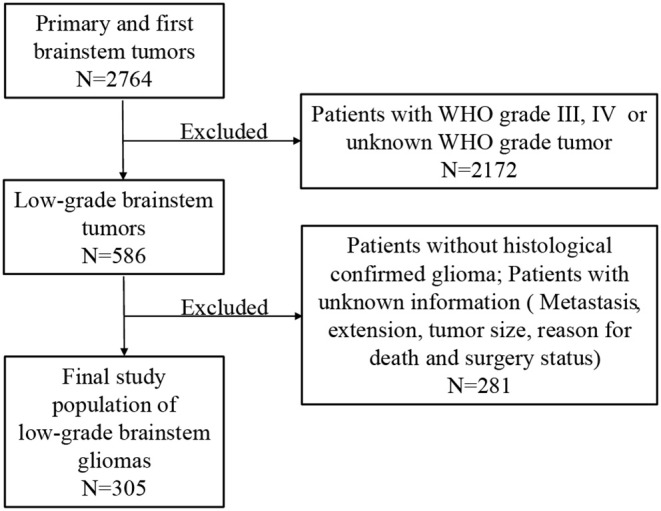
Flowchart of low-grade brainstem glioma patient selection.

The SEER database, which is maintained by the National Cancer Institute, was searched to identify data deposited between 2004 and 2015. The SEER database provides prospectively collected data on patients with deidentified information. For this reason, no approval was required from the institutional review board for this study. All patients with first and primary brainstem tumor were included. Patients with WHO grade III or IV or unknown WHO grade tumors were excluded. Patients without histologically confirmed glioma and other crucial variates (metastasis, extension, tumor size, reason for death, and surgery status) were also excluded (Figure 1).

### Covariates Included

The following patient data were obtained for the analysis: age at diagnosis (patients aged <22 years were assigned to the pediatric group, and those aged ≥22 years were assigned to adult group), sex, race (white, black, American Indian/Alaska Native, Asian/Pacific Islander), marital status (married, unmarried), WHO grade (I, II), surgery [unknown surgery status, local excision/biopsy, subtotal resection, gross total resection (GTR)], tumor size (size ≤ 3.6 cm, size >3.6 cm, the best cutoff value was defined according to X-tile software), metastasis (yes, no), RT and CT (both, none/unknown, RT, CT), and extension pattern (brainstem, cerebellum, ventricular, and other categories). We divided the histological type into diffuse astrocytic and oligodendroglial tumors (DAOTs), other astrocyte tumors (OATs), and ependymal tumors (ETs) according to the 2016 WHO classification of tumors of the central nervous system (Table 1).

**Table 1:** Patient demographics, tumor characteristics, and treatment options of 305 low-grade brainstem glioma patients.

**Characteristic**	**ALL *n* (%)**	**Pediatric *n* (%)**	**Adult *n* (%)**	***P***
Population size	305 (100)	165 (54.1)	140 (45.9)	
Age, median (range), y		8.31 (<22)	46.01 (22–85)	
Era of diagnosis				0.353
2004–2009	134 (43.9)	77 (25.2)	57 (18.7)	
2010–2015	171 (56.1)	88 (28.9)	83 (27.2)	
Sex				0.500
Female	132 (42.3)	68 (22.3)	64 (20.0)	
Male	173 (56.7)	97 (31.8)	76 (24.9)	
Race				0.565
Asian/Pacific Islander	17 (5.6)	9 (3.0)	8 (2.6)	
Black	27 (8.9)	12 (3.9)	15 (5.0)	
White	261 (85.5)	144 (47.2)	117 (38.3)	
Marital status				
Unmarried	224 (73.4)	164 (53.8)	60 (19.6)	<0.001^†^
Married	81 (26.6)	1 (0.3)	80 (26.3)	
WHO grade				
I	137 (44.9)	98 (32.1)	39 (12.8)	<0.001^†^
II	168 (55.1)	67 (22.0)	101 (33.1)	
Surgery				0.988
Local excision/biopsy	60 (19.7)	32 (10.5)	28 (9.2)	
STR	65 (21.3)	35 (11.5)	30 (9.8)	
GTR	180 (59.0)	98 (32.1)	82 (26.9)	
Size				
≤ 3.6 cm	158 (51.8)	66 (21.6)	92 (30.2)	<0.001^†^
>3.6 cm	147 (48.2)	99 (32.5)	48 (15.7)	
Metastasis				
Yes	8 (2.6)	4 (1.3)	4 (1.3)	1.000
No	297 (97.4)	161 (52.8)	136 (46.6)	
Adjuvant therapy				
Both	18 (5.9)	14 (4.6)	4 (1.3)	<0.001^†^
Radiotherapy	100 (32.8)	42 (13.7)	58 (19.1)	
Chemotherapy	25 (8.2)	24 (7.9)	1 (0.3)	
None/unknown	162 (53.1)	85 (27.9)	77 (25.2)	
Extension				
Brainstem	120 (39.4)	75 (24.6)	45 (14.8)	<0.001^†^
Cerebellum	25 (8.2)	15 (4.9)	10 (3.3)	
Ventricular	105 (34.4)	43 (14.1)	62 (20.3)	
Other	55 (18.0)	32 (10.5)	23 (7.5)	
Cancer-specific death status				
Alive	276 (90.5)	155 (50.8)	121 (39.7)	0.042^†^
Dead	29 (9.5)	10 (3.3)	19 (6.2)	
Vital status				
Alive	265 (86.8)	152 (49.8)	113 (37.0)	0.006^†^
Dead	40 (13.2)	13 (4.3)	27 (8.9)	
Histology				
DAOT	31 (10.2)	19 (6.2)	12 (4.0)	<0.001^†^
ET	138 (45.2)	45 (14.8)	93 (30.4)	
OAT	136 (44.6)	101 (33.1)	35 (11.5)	

† *P < 0.05, statistically significant*.

### Statistical Analyses

To analyze all different prognostic variables associated with the CSS and OS, both univariate and multivariate cox proportional hazard models were applied to calculate hazard ratios (HRs) and the corresponding 95% confidence intervals (CIs). *P* < 0.05 was considered statistically significant. And Kaplan–Meier curves and decision tree were plotted to compare the CSS of patients with LGBSG patients by all different prognostic factors. The study population was randomly divided into training group (*n* = 152) and test group (*n* = 153). All different variables were further identified by L1-penalized (Lasso) regression model. The risk scores were then calculated according to the formula, risk score = β1X1+ β2X2 + … + βnXn (β, regression coefficient; X, prognostic factors). Then a nomogram was developed using the package of RMS in R version 3.5.1 (http://www.r-project.org/). A calibration curve was used for internal validation, which described the average predictive value against actual observation and evaluated the performance of nomogram visually. Harrell's concordance index (C-index) and time-dependent receiver operating characteristic (ROC) curve were used to evaluate the discrimination of nomogram to assess the consistency between the actual and predicted CSS rate. The clinical use of nomogram was estimated by decision curve analysis (DCA), which is a novel method that estimates predictive models from the perspective of clinical consequences.

## Results

### Patient Population and Baseline Characteristics

A total of 305 LGBSG patients were analyzed. Among them, 165 were pediatric with a mean age of 8.31 years (<22 years), and 140 were adult with a mean age of 46.01 years (22–85 years). The data showed that LGBSG had a slight male preponderance (56.7%), but it was not statistically significant (*P* = 0.500). At the time of data collection, the CSS rates for pediatric and adults patients were 93.9 and 86.4%, respectively (*P* = 0.042). Majority of patients were white (*n* = 261, 85.5%), whereas 27 (8.9%) were black, and 17 (5.6%) were Asian/Pacific Islander. The majority of patients were diagnosed during 2010–2015 compared to 2004–2009 (*n* = 171, 56.7% vs. *n* = 134, 43.9%). At the time of data collection, 265 patients (86.8%) were alive, whereas 40 cases (13.2%) had died. Of the 40 deaths, 29 died of cancer-specific events.

Metastasis occurred in eight patients (2.6%), including four pediatric and four adult patients, indicating no difference (*P* = 1.000). In contrast, there were significant differences between pediatric and adult patients in tumor size; the proportion of patients with tumor size >3.6 cm in the adult groups was significantly lower than that in pediatric patients (34.3 vs. 60.0%, *P* < 0.001). In terms of tumor extension pattern, it showed significant difference between the two groups (*P* < 0.001); the proportion of patients with tumor extended to ventricular system in adults was significantly higher than that in pediatric patients (44.3 vs. 26.1%, *P* < 0.001). Concerning treatment options, the extent of surgery was not significantly different between the two groups (*P* = 0.988). But for adjuvant therapy, notable differences were observed between the two groups; more adult patients received RT (41.4 vs. 25.5%), and fewer adult patients received CT (0.1 vs. 14.5%) (*P* < 0.001) (Table 1).

### Prognostic Factors of CSS and OS

The univariate analysis showed that the pediatric group (HR, 0.39; 95% CI, 0.18–0.84; *P* = 0.016), GTR (HR, 0.28; 95% CI, 0.11–0.69; *P* = 0.005), and extension to ventricular system (HR, 0.36; 95% CI, 0.13–0.99; *P* = 0.049) were associated with increased CSS rate (*P* < 0.05). In contrast, WHO grade II (HR, 2.69 (1.15–6.30 *P* < 0.001), DAOT (HR, 5.65; 95% CI, 2.45–13.08; *P* < 0.001), and RT (HR, 2.46; 95% CI, 0.46–9.71; *P* = 0.028) were significantly associated with decreased CSS rate (*P* < 0.05). In multivariate analysis, pediatric group (HR, 0.28; 95% CI, 0.10–0.76; *P* = 0.012) and ventricular system involvement (HR, 0.40; 95% CI, 0.14–1.20; *P* = 0.010) were independently associated with improved CSS rate. On the contrary, metastasis (HR, 5.20; 95% CI, 1.03–26.38; *P* = 0.046) and DAOT (HR, 5.14; 95% CI, 1.72–15.39; *P* = 0.003) were independently associated with decreased CSS rate. Analysis of surgical procedures showed that GTR (HR, 0.40; 95% CI, 0.15–1.12; *P* = 0.081) was not significantly associated with better CSS after adjusting for confounding effects of each variable when compared with biopsy group. World Health Organization grade II (HR, 2.57; 95% CI, 0.30–21.75; *P* = 0.386) also lost its significance in multivariate analysis (Table 2). The predictive factors of OS were similar, which were only slightly different from those of CSS in HR, 95% CI, and *P*-value (Table S1).

**Table 2:** Univariate and multivariate Cox proportional hazard regression analyses to determine prognostic factors of cancer-specific survival for patients with low-grade brainstem glioma.

	**5-y CSS rate (%)**	**10-y CSS rate (%)**	**Univariate analysis**		**Multivariate analysis**	
			**HR (95% CI)**	***P***	**HR (95% CI)**	***P***
Age						
≥22 Adult	84.9	74.7	1 [Reference]		1 [Reference]	
<22 Pediatric	93.2	86.6	0.39 (0.18–0.84)	0.016^†^	0.28 (0.10–0.76)	0.012^†^
Sex						
Female	88.1	83.5	1 [Reference]		1 [Reference]	
Male	90.7	90.7	0.72 (0.35–1.49)	0.375	0.96 (0.42–2.17)	0.918
Race						
White	90.0	7.6	1 [Reference]		1 [Reference]	
Black	83.5	83.5	1.62 (0.56–4.66)	0.374	1.42 (0.46–4.43)	0.545
Asian/Pacific Islander	90.0	/	0.73 (0.10–5.37)	0.753	1.41 (0.18–11.22)	0.744
Marital status						
Unmarried	88.1	93.6	1 [Reference]		1 [Reference]	
Married	85.0	86.2	1.02 (0.45–2.31)	0.956	0.54 (0.20–1.46)	0.226
Grade						
I	93.6	93.6	1 [Reference]		1 [Reference]	
II	86.2	82.8	2.69 (1.15–6.30)	<0.001^†^	2.57 (0.30–21.75)	0.386
Surgery						
Local excision/biopsy	81.3	81.3	1 [Reference]		1 [Reference]	
STR	79.1	/	1.22 (0.50–2.97)	0.659	1.64 (0.61–4.40)	0.570
GTR	95.5	93.1	0.28 (0.11–0.69)	0.005^†^	0.40 (0.15–1.12)	0.081
Size						
≤ 3.6 cm	88.4	86.2	1 [Reference]		1 [Reference]	
>3.6 cm	90.6	88.9	0.82 (0.39–1.70)	0.591	1.26 (0.49–3.25)	0.634
Metastasis						
No	90.0	87.8	1 [Reference]		1 [Reference]	
Yes	68.6	/	2.75 (0.65–11.57)	0.168	5.20 (1.03–26.38)	0.046^†^
Adjuvant therapy						
None/unknown	93.9	92.0	1 [Reference]		1 [Reference]	
Radiotherapy	81.6	81.6	2.46 (0.46–9.71)	0.028^†^	1.48 (0.57–3.82)	0.421
Chemotherapy	94.4	78.7	1.37 (0.30–6.27)	0.683	0.81 (0.14–4.50)	0.806
Both	86.2	/	2.12 (0.46–9.71)	0.333	0.93 (0.16–5.36)	0.938
Extension						
Brainstem	85.0	83.0	1 [Reference]		1 [Reference]	
Cerebellum	92.0	92.0	0.58 (0.13–2.51)	0.462	0.64 (0.13–3.16)	0.581
Ventricular	95.4	92.2	0.36 (0.13–0.99)	0.049^†^	0.40 (0.14–1.20)	0.010^†^
Other	87.4	87.4	0.85 (0.33–2.16)	0.727	0.91 (0.30–2.83)	0.877
Histology						
ET	92.1	88.2	1 [Reference]		1 [Reference]	
OAT	93.6	93.6	0.64 (0.25–1.65)	0.358	1.83 (0.20–17.33)	0.596
DAOT	59.1	59.1	5.65 (2.45–13.08)	<0.001^†^	5.14 (1.72–15.39)	0.003^†^

† *P < 0.05, statistically significant*.

**Figure 2:**
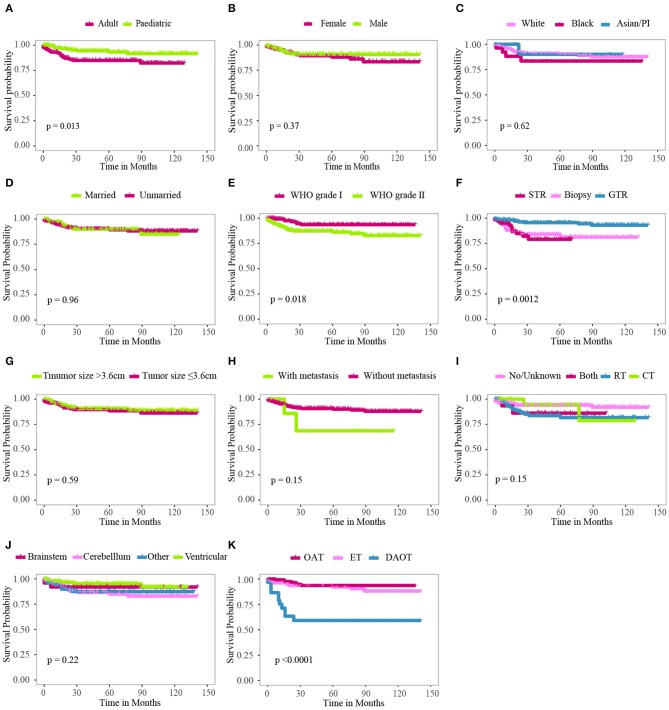
Kaplan–Meier curves for patients with LGBSG by different variates. **(A)** Age group, **(B)** sex, **(C)** race, **(D)** marital status, **(E)** WHO grade, **(F)** surgery, **(G)** tumor size, **(H)** metastasis, **(I)** adjuvant therapy, **(J)** tumor extension, **(K)** histology.

**Figure 3:**
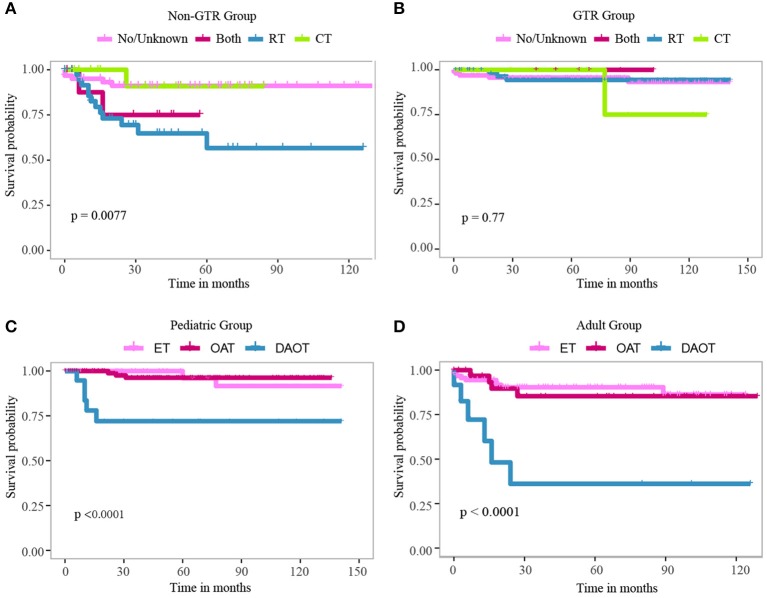
Kaplan–Meier curve for patients with LGBSG in non-GTR group **(A)** and GTR group **(B)** treated with different adjuvant therapies. Kaplan–Meier curve for pediatric patients **(C)** and adult patients **(D)** with different histology types.

**Figure 4:**
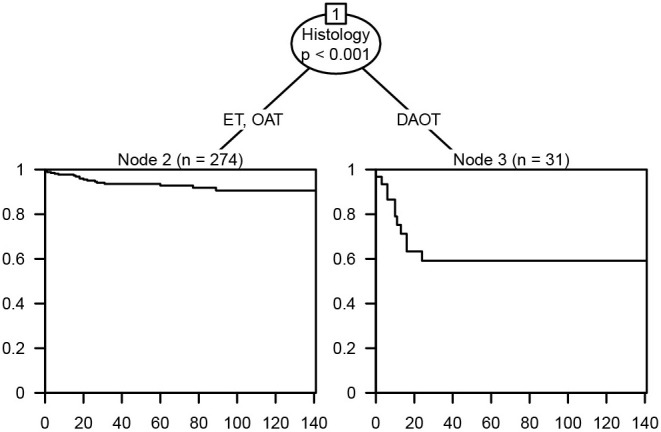
Decision tree for the management of low-grade brainstem glioma patient.

Kaplan–Meier curves were plotted to compare the CSS of LGBSG patients by different variates. The result showed that age group (*P* = 0.013), WHO grade (*P* = 0.018), surgery (*P* = 0.0012), and histology (*P* < 0.0001) showed significant difference (Figure 2). To assess the benefit of adjuvant therapy in the non-GTR group, one additional Kaplan–Meier curve was plotted. The result showed that patients who received RT or RT combined with CT had worse survival (*P* = 0.0077) (Figure 3). The decision tree identified that histological-type DAOT (*P* < 0.001) was the most distinguishable factor for survival period (Figure 4).

### Prognostic Nomogram for CSS and OS

**Figure 5:**
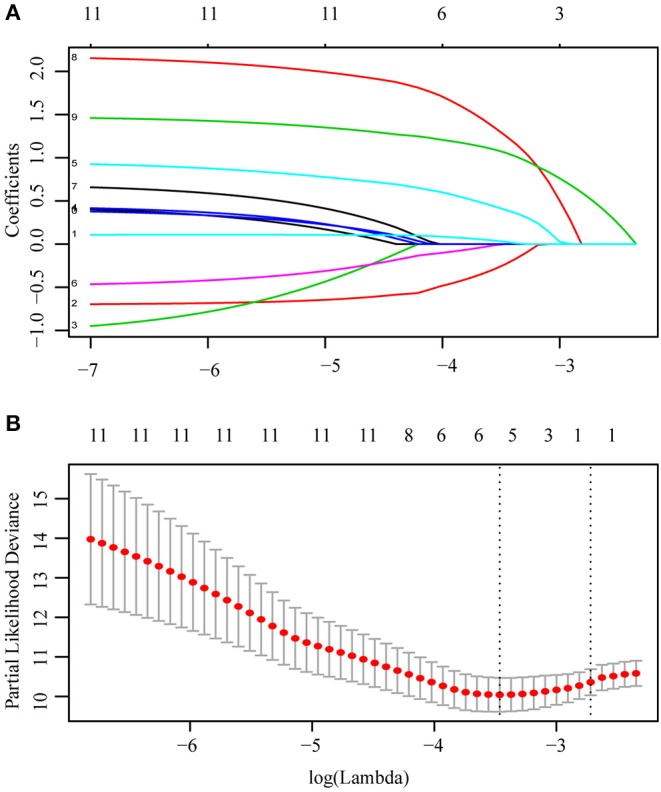
L1-penalized (Lasso) regression model were applied to further identified prognostic factors in training cohort. Race, surgery, histology, and adjuvant therapy were identified for CSS **(A)**. LASSO coefficient profiles of the features **(B)**. Ten-time cross-validation for tuning paremeter selection in the Lasso Model.

**Figure 6:**
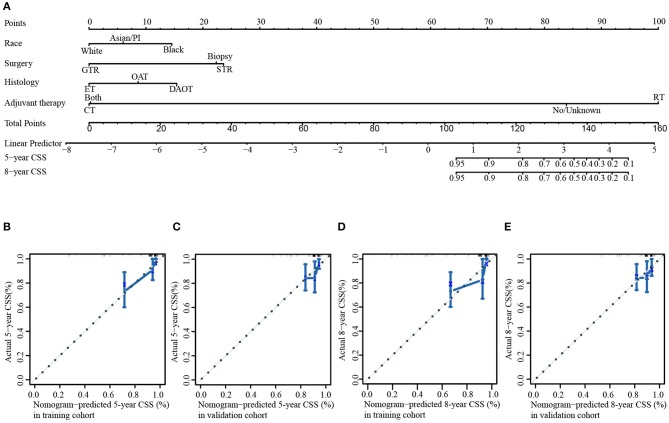
Nomogram and internal calibration for cancer-specific survival rate. **(A)** Nomogram to predict 5- and 8-year CSS rates of low-grade brainstem glioma patients. The internal calibration curve to predict 5-year CSS rate in training cohort **(B)** and validation cohort **(C)**. The internal calibration curve to predict 8-year CSS rate in training cohort **(D)** and validation cohort **(E)**.

**Figure 7:**
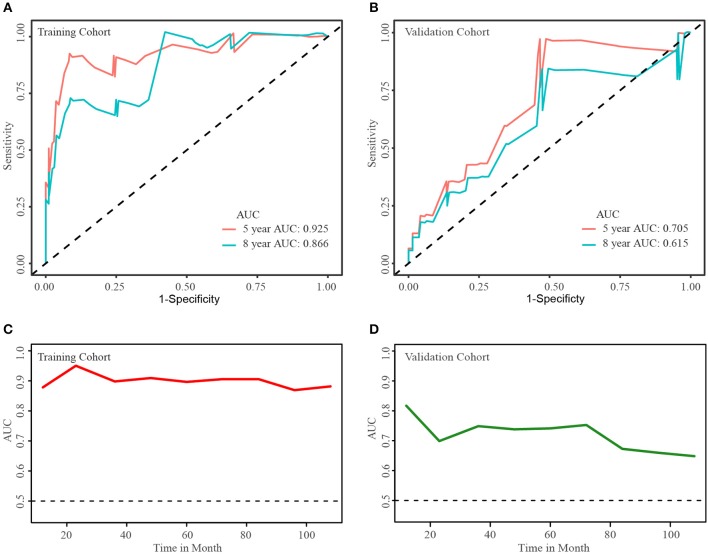
Time-dependent ROC curve and areas under ROC curve at different time points. Areas under ROC curve of 5- and 8-year cancer-specific survival rates in training cohort **(A)** and validation cohort **(B)**. Areas under ROC curve at different time points in training cohort **(C)** and validation cohort **(D)**.

**Figure 8:**
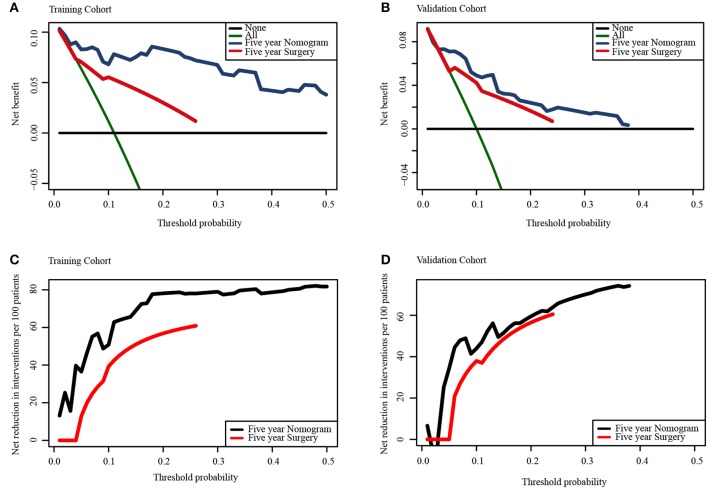
Decision curve analysis for the nomogram and the model including surgery only in the prediction of the cancer-specific survival rates of patients at 5-year point in training cohort **(A,C)** and validation cohort **(B,D)**.

The population was randomly divided into training group (*n* = 152) and validation group (*n* = 153) (Table 3). We applied L1-penalized (Lasso) regression model to further identify prognostic factors for the CSS (Figure 5) and OS (Figure S1) of LGBSG patients. Race, surgery, histology, and adjuvant therapy were incorporated into the nomogram for CSS (Figure 6). And metastasis, age group, histology, adjuvant therapy, and WHO grade were incorporated into the nomogram for OS (Figure S2). The calibration curve for the probability of postoperative CSS (Figure 6) and OS (Figure S2) at 5- and 8-year showed that there was a good consistency between the predicted survival probability and the actual survival probability in the data set. The C-index of internal validation for CSS and OS prediction was 0.87 (95% CI, 0.78–0.96) and 0.78 (95% CI, 0.70–0.86), respectively. The time-dependent ROC curve and area under curve (AUC) were established. Generally, the AUCs for CSS (Figure 7) and OS (Figure S3) at different time points in training and validation cohort were ~0.7, which suggested the nomogram was accurate and effective at different time points. The clinical use was evaluated by DCA; the 5-year DCA curves in training and validation cohort for CSS nomogram (Figure 8) and OS nomogram (Figure S4) yield larger net benefits than the model including surgery only.

**Table 3:** Training and validation cohort for nomogram to predict 5- and 8-year cancer-specific survival rates of low-grade brainstem glioma patient.

	**Training *n* (%)**	**Validation *n* (%)**	***P***
Age			1.000
≥22 Adult	70 (23.0)	70 (23.0)	
<22 Pediatric	82 (26.9)	83 (27.1)	
Sex			0.448
Female	62 (20.3)	70 (23.0)	
Male	90 (29.5)	83 (27.2)	
Race			0.199
White	126 (41.3)	135 (44.3)	
Black	14 (4.6)	13 (4.3)	
Asian or Pacific Islander	12 (3.9)	5 (1.6)	
Marital status			0.417
Unmarried	108 (35.4)	116 (38.0)	
Married	44 (14.4)	37 (12.2)	
WHO grade			0.272
I	63 (20.7)	74 (24.3)	
II	89 (29.2)	79 (25.8)	
Surgery			0.399
Local excision/biopsy	34 (11.2)	26 (8.5)	
STR	29 (9.5)	36 (11.8)	
GTR	89 (29.2)	91 (29.8)	
Size			0.862
≤ 3.6 cm	80 (26.2)	78 (25.6)	
>3.6 cm	72 (23.6)	75 (24.6)	
Metastasis			0.287
No	150 (49.2)	147 (48.2)	
Yes	2 (0.6)	6 (2.0)	
Adjuvant therapy			0.920
No/unknown	83 (27.2)	79 (25.9)	
Both	9 (3.0)	9 (3.0)	
Radiotherapy	49 (16.0)	51 (16.7)	
Chemotherapy	11 (3.6)	14 (4.6)	
Extension			0.731
Brainstem	61 (22.0)	59 (19.3)	
Cerebellum	14 (4.6)	11 (3.6)	
Ventricular	53 (17.4)	52 (17.0)	
Other	24 (7.9)	31 (10.2)	
Cancer-specific death event			1
Alive	138 (45.3)	138 (45.2)	
Dead	14 (4.6)	15 (4.9)	
Vital status			0.595
Alive	130 (42.6)	135 (44.3)	
Dead	22 (7.2)	18 (5.9)	

## Discussion

Marked differences were observed in the epidemiological and biological characteristics of pediatric and adult patients with LGBSG. The multivariate analysis showed that the pediatric group was a significant and independent predictor of better OS and CSS. At the time of data collection, the CSS rates for pediatric and adult patients were 93.9 and 86.4% (*P* = 0.042), respectively. For pediatric patients with LGBSG, one retrospective study carried out at the Mayo clinic including 48 pediatric patients with LGBSG showed a median OS of 177.6 months and 5-year OS rate of 67% (21). Another retrospective study of 52 pediatric focal LGBSG patients reported a 5-year OS rate of 98% (20). For adult LGBSG patients, Reithmeier et al. (6) reported that the median OS for 30 WHO grade II BSG patients was 26.2 months, and Kesari et al. (22) reported that the median OS for 16 WHO grade I patients was 83 months. Generally, the results of other clinical studies support our conclusion that pediatric patients have a better survival than adult patients. In this present cohort, our analysis showed that more pediatric patients had tumors >3.6 cm, and more adult patients had tumors involving the ventricular system, which had not been reported by other studies yet.

To investigate the effect of tumor characteristics on patient survival, we performed univariate and multivariate analyses and plotted Kaplan–Meier curves and decision tree. Our analysis revealed that WHO grade II was independently associated with worse CSS and OS in univariate analysis. The Kaplan–Meier curve also showed that the patients with WHO grade II tumor had worse survival rate. In this cohort, the 10-year CSS rates of patients with WHO grade I tumor and with WHO grade II tumor were 55.1 and 44.9%, respectively. Consistently, Ahmed et al. (21) reported that the 5-year OS rate of patients with WHO grade I tumors was significantly higher than that of patients with WHO grade II tumors in a study with 48 pediatric LGBSG patients (71 vs. 52%, *P* = 0.08). In clinical studies investigating adult BSG patients, those with WHO grade I tumors exhibited better survival rate than patients WHO grade II tumor (6). In addition, we divided the histological type into DAOT, OAT, and ET according to the 2016 WHO classification of tumors of central nerves system. And data analysis showed that DAOT was an important predictor for worse survival. The 10-year CSS rate of patients with DAOT and ET were 59.1 and 88.2%, respectively. This phenomenon had not been reported by other groups yet, which gave us a new insight about LGBSG.

Surgical treatment of the brainstem tumor has often been considered to be a difficult operation due to its critical position and crucial fundamental function. However, the advances in neuroimaging techniques (high-resolution MRI), anesthesia and neurosurgery have rendered resection of brainstem tumors feasible. Based on our analysis, GTR was significantly associated with better CSS and OS in univariate analysis when compared with patients treated with biopsy, but lost its significance in multivariate analysis. And this might be caused by the small sample size analyzed (*n* = 180) as we did not include the patients with unknown surgery status. The Kaplan–Meier curve confirmed that the patients receiving GTR had the highest survival rate (*P* = 0.0012). The 10-year CSS rates for patients treated with GTR and biopsy were 93.1 and 81.3%, respectively. So far, several studies have been reported concerning partial and even complete resection of brainstem tumor, and clinical outcomes in such studies are positive (23–25). A study conducted by Mayo Clinic on pediatric LGBSG patients concluded that tumor resection vs. biopsy only improved patient survival with statically increased 5-year OS rate (85 vs. 50%, *P* = 0.002) (21). Teo and Siu (23) reported a 100% 5-year OS rate of 23 pediatric LGBSG patients treated with endoscope-assisted microsurgery. Lundar et al. (14) performed resections on 15 pediatric patients diagnosed with low-grade midbrain glioma. They reported prolonged survival period of the patients. This study also found that tumor extension to ventricular system was a significant predictor of CSS by univariate and multivariate analyses. A population-based study focusing on HGBSG also concluded that ventricular system involvement may increase patient survival at 9 months compared with those with tumors confined to the brainstem (9). And this might be because tumors involving the ventricular system are more amenable to surgical resection. Although there is high heterogeneity that existed within different clinical studies, we could make a conclusion that most of the LGSBG patients benefited from GTR with prolonged survival. On the basis of the present analysis and results from other clinical studies (Table 4), it is considerable to suggest the safe maximal surgical resection as an effective treatment for LGBSG patients.

**Table 4:** Studies reporting prognostic factors of pediatric low-grade brainstem glioma patients.

**References**	**Patient included**	**Patient number**	**Median follow-up (mo)**	**Prognostic factors**
Sandri et al. (13)	Focal BSG	17	25	GTR
Fried et al. (15)	LGBSG	96	57.6	RT and CT was not associated with OS
Klimo et al. (20)	Focal LGBSG	52	120	GTR, intrinsic tumor
Ahmed et al. (21)	LGBSG	48	177.6	GTR, WHO grade, diffuse tumor
Lundar et al. (14)	Low-grade midbrain glioma	15	96	GTR
Upadhyaya et al. (12)	LGBSG	23	106	Combination of surgery, RT and CT

Gross total resection plays an important role in the management of LGBSG patients and is considered to be a favorable predictor of better CSS and OS. However, for patients undergoing STR or biopsy only with residual lesion, there is no consensus as to whether they benefit from upfront postoperative adjuvant therapy (RT/CT). Generally, our results show that more adult LGBSG patients received RT, whereas more pediatric LGBSG patients were treated with CT for adjuvant therapy. This is because that RT has been reported to cause neurocognitive deficits and academic achievement problems in pediatric patients (26). Surprisingly, the univariate analysis results showed that RT was associated with worse CSS and OS, but this effect was lost after the correction of multivariate analysis. At the same time, Kaplan–Meier curve showed that adjuvant therapy added no benefits in patients with GTR and non-GTR. Ahmed et al. (21) also concluded that postoperative RT was associated with decreased OS based on univariate analysis. Given that RT was preferentially performed in adult patients or those who received no surgery and biopsy only was an important reason, this selection bias may account for this effect. Furthermore, univariate analysis showed that CT was not significantly associated with CSS and OS. Indeed, no CT regimen has been proven to be effective for LGBSG patients. Only few cases were reported to have a good response to CT (14, 27, 28). In addition, the Kaplan–Meier curves plotted both in all patients (*n* = 305) and non-GTR group (*n* = 125) supported that adjuvant therapy provided no clinical benefits. A clinical study including 96 pediatric patients with LGBSG reported that upfront adjuvant therapy (RT/CT) did not significantly improve the prognosis of patients with residual tumor compared with observation only (15). In summary, upfront adjuvant therapy is not beneficial to CSS and OS. Therefore, observation may be a safe alternative for LGBSG patients receiving STR or biopsy only with residual lesion.

This study has the following limitations. Although the SEER database contains a large number of records, it lacks other important information. For instance, data about the concrete position and growth pattern of tumor were not accessible in SEER database, which were important for tumor categorization and evaluating the prognosis of patients with LGBSG (20, 21, 29). Moreover, other clinical features of patients including functional status and neurologic symptoms were not available, which were reported to be predictive factors of patient prognosis (30–32). In addition, the surgical approach, radiation dosage, and CT protocol were not included in our analysis, although these factors influence the patient prognosis. And we do not know the detail information about how the tumor size was measured. Another weakness of this study is that there are 61 patients with specific histological diagnosis having the record of “no surgery” in the SEER database, which is confusing. So we did not include these 61 patients in the data set so as to make reliable conclusion. Given that the surgical experience is growing and efforts are made to attempt preferable adjuvant therapy methods (16, 33–35), the present study based on cases reported between 2004 and 2015 could not capture the most updated clinical evidence.

The strengths of this study include being the largest population-based real world study about LGBSG from SEER database. This study confirmed many findings in other single-center small dataset clinical studies with increased strength and low bias. This study describes, for the first time, the characteristics and prognostic factors of adult patients with LGBSG and compared the differences between pediatric and adult patients with LGBSG. Moreover, an accurate and effective nomogram was established to predict CSS and OS rate of LGBSG patients.

## Conclusion

This population-based real world study of 165 pediatric and 140 adult LGBSG patients demonstrates that there are differences between these two groups. And safe maximal surgical resection was suggested as an effective treatment according to our data analysis and other clinical studies, yet with caution surgical resection may result in significant neurological deficits. Observation seems to be optional for patients with residual tumor after incomplete surgical resection because upfront adjuvant therapy had no effect on CSS according to the analysis. This study provides valuable data highlighting the need for prospective clinical studies in order to validate outcomes.

## Data Availability Statement

The data of LGBSG patients searched in SEER database are freely available.

## Author Contributions

QC and SF made substantial contribution to the design of this study. ZhuL and QC carried out the analysis and interpreted the data. ZhuL and SF made contributions to the drafting of the manuscript. JL and HC made contributions to the review of previous literature. JH, FF, and LC contributed substantially to the revision of the manuscript. QC and ZhiL made substantial contributions to the conception of the manuscript, and were responsible for the quality of the overall manuscript. All authors approved the final version of the manuscript.

### Conflict of Interest

The authors declare that the research was conducted in the absence of any commercial or financial relationships that could be construed as a potential conflict of interest.
