# Supplementary material for: The Epidemiological Characteristics and Prognostic Factors of Low-Grade Brainstem Glioma: A Real-World Study of Pediatric and Adult Patients
Source: Front Oncol. 2020 Apr 9;10:391. doi: 10.3389/fonc.2020.00391 (PMC7160332; doi:10.3389/fonc.2020.00391)
Supplement: Supplementary file 1 [file Data_Sheet_1.pdf]

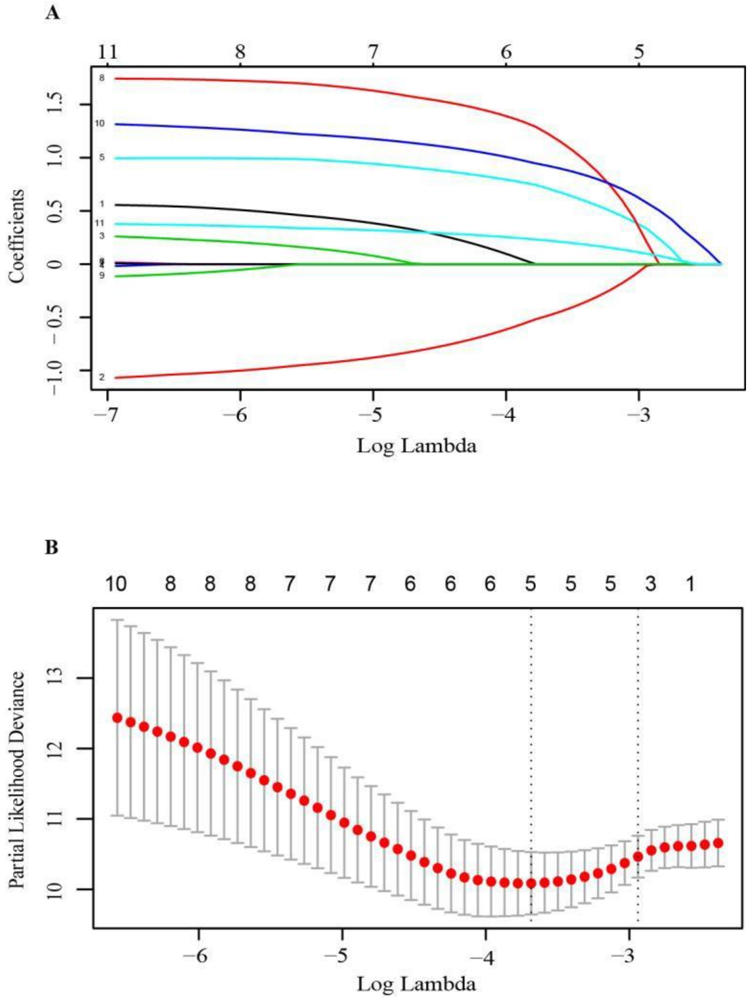

Figure S1 L1-penalized (Lasso) regression model were applied to further identified prognostic factors in training cohort. Metastasis, age group, histology, adjuvant therapy and WHO grade were identified for OS. (A). LASSO coefficient profiles of the features. (B). Ten-time cross-validation for tuning paremeter selection in the Lasso Model.

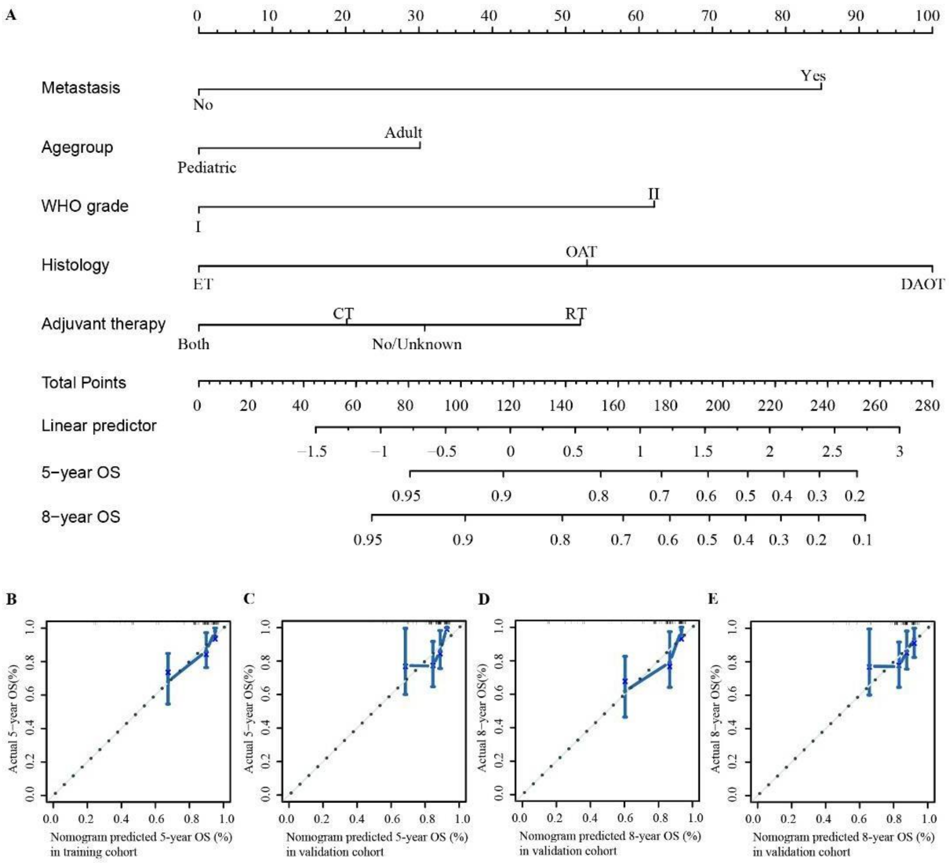

Figure S2 Nomogram and internal calibration for overall survival rate.**(A)** Nomogram to predict 5-year and 8 year OS rates of low-grade brainstem glioma patients; The internal calibration curve to predict 5-year OS rate in training cohort **(B)** and validation cohort **(C)**; The internal calibration curve to predict 8-year OS rate in training cohort **(D)** and validation cohort **(E)**.

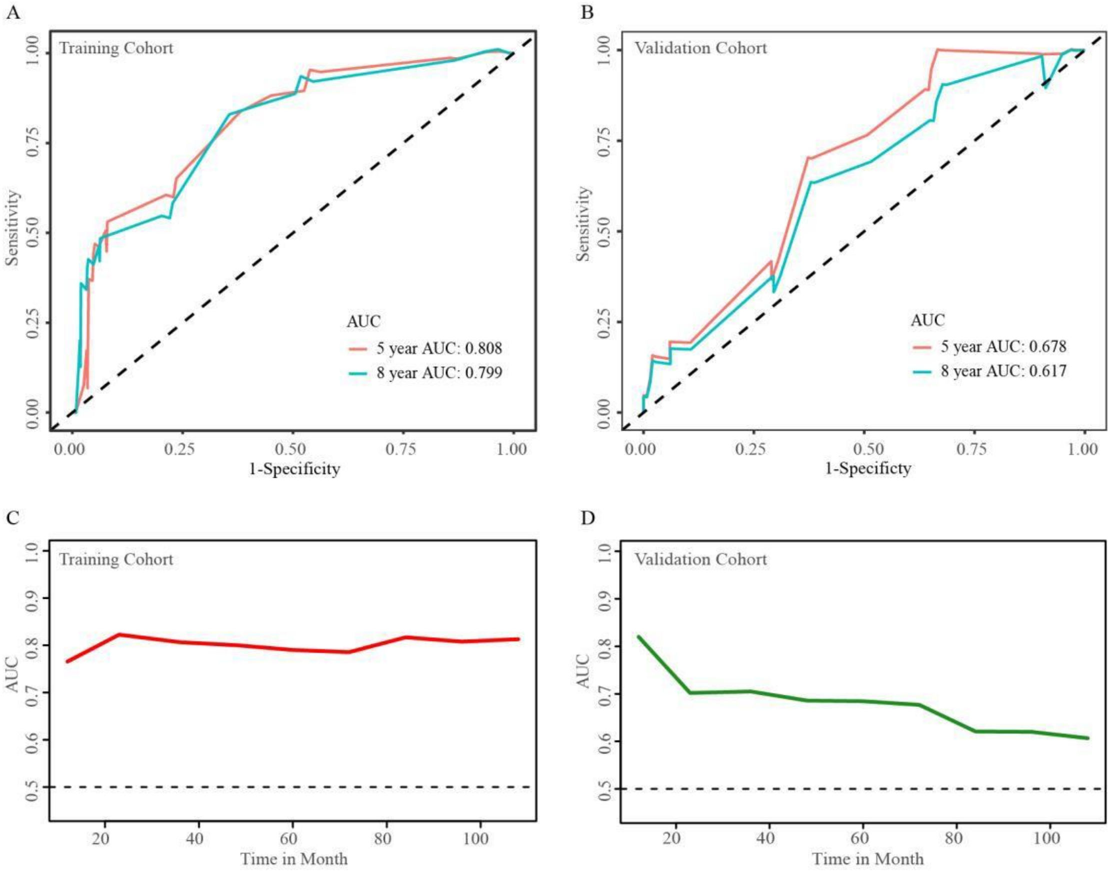

Figure S3 Time-dependent ROC curve and areas under ROC curve at different time points. Areas under ROC curve of 5-year and 8-year overall survival rates in training cohort **(A)** and validation cohort **(B);** Areas under ROC curve at different time points in training cohort **(C)** and validation cohort **(D);**

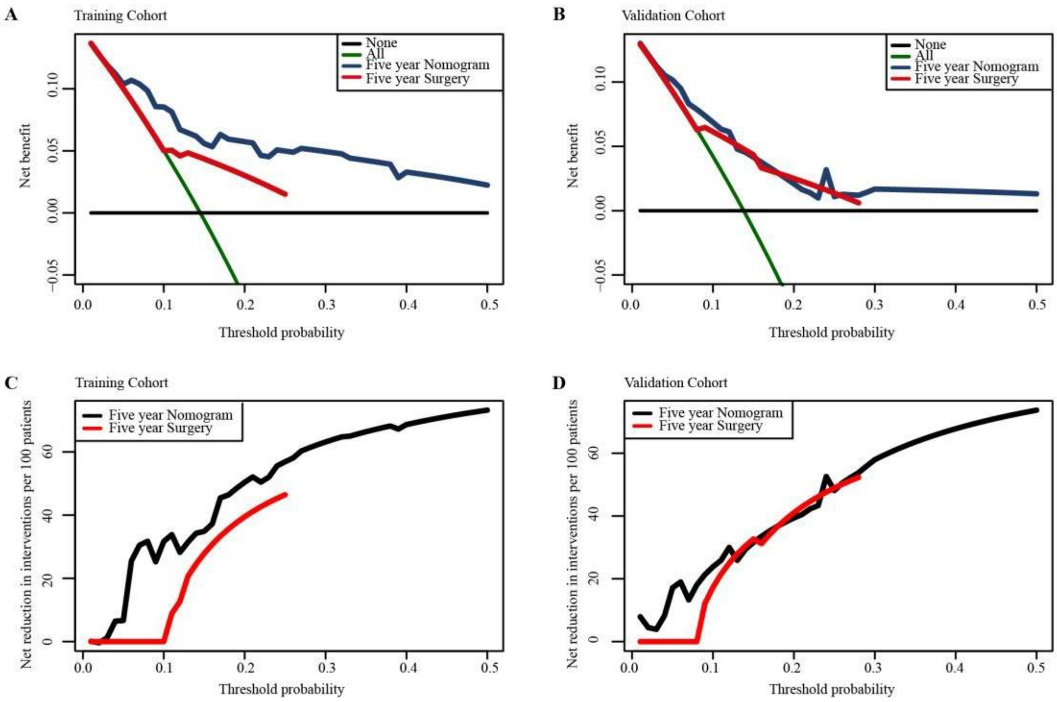

Figure S4 Decision curve analysis for the nomogram and the model including surgery only in prediction of the overall survival rates of patients at 5 year point in training (A, C) and validation (B, D) cohort.

Table S1 Univariate and multivariate cox proportional hazard regression analyses to determine prognostic factors of overall survival for patients with low-grade brainstem glioma

	5-year OS rate (%)	10-year OS rate (%)	Univariate analysis		Multivariate analysis	
			HR (95%CI)	P-value	HR (95%CI)	P-value
Age						
>21 Adult	79.1	74.9	1 [Reference]		1 [Reference]	
≤21 Pediatric	91.3	89.8	0.36 (0.18-0.69)	0.002†	0.31 (0.13-0.73)	0.008†
Sex						
Female	84.1	77.8	1 [Reference]		1 [Reference]	
Male	87.2	87.2	0.70 (0.37-1.29)	0.252	0.85 (0.43-1.70)	0.647
Race						
White	86.7	83.2	1 [Reference]		1 [Reference]	
Black	83.5	83.5	1.18 (0.42-3.32)	0.760	1.04 (0.35-3.11)	0.941
Asian/PI	73.3	/	1.60 (0.49-5.22)	0.437	2.69 (0.77-9.45)	0.121
Marital status						
Unmarried	86.2	85.0	1 [Reference]		1 [Reference]	
Married	85.0	77.2	1.30 (0.67-2.51)	0.442	0.70 (0.31-1.56)	0.384
WHO grade						
I	92.1	92.1	1 [Reference]		1 [Reference]	
II	80.8	76.4	2.96 (1.14-6.21)	0.004†	2.36 (0.42-13.30)	0.330
Surgery						
Biopsy	78.2	78.2	1 [Reference]		1 [Reference]	
STR	76.1	/	1.10 (0.48-2.52)	0.814	1.46 (0.60-3.56)	0.410
GTR	91.2	87.8	0.44 (0.21-0.92)	0.028†	0.58 (0.26-1.32)	0.193
Size						
≤3.6cm	84.0	80.3	1 [Reference]		1 [Reference]	
>3.6cm	87.8	86.1	0.74 (0.40-1.39)	0.353	1.18 (0.54-2.58)	0.683
Metastasis						
No	86.3	83.4	1 [Reference]		1 [Reference]	
Yes	68.6	/	1.97 (0.47-8.15)	0.352	3.55 (0.77-16.29)	0.104
Adjuvant therapy						
None/Unknown	89.7	87.8	1 [Reference]		1 [Reference]	
Radiotherapy	77.6	75.3	2.05 (1.06-3.96)	0.032†	1.29 (0.60-2.79)	0.519
Chemotherapy	94.4	78.7	0.86 (0.20-3.74)	0.839	0.78 (0.16-3.79)	0.758
Both	86.2	/	1.32 (0.30-5.74)	0.714	0.87 (0.17-4.40)	0.866
Extension						
Brainstem	80.3	76.5	1 [Reference]		1 [Reference]	
Cerebellum	88.0	88.0	0.63 (0.19-2.09)	0.447	0.59 (0.16-2.19)	0.432
Ventricular	91.2	88.1	0.48 (0.22-1.03)	0.061	0.45 (0.19-1.06)	0.067
Other	87.4	87.4	0.62 (0.25-1.52)	0.293	0.64 (0.23-1.79)	0.396
Histology						

ET	85.6	81.9	1 [Reference]		1 [Reference]	
OAT	92.0	92.0	0.48 (0.22-1.05)	0.067	1.29 (0.21-7.19)	0.781
DAOT	59.1	51.7	3.60 (1.75-7.44)	<0.001†	3.66(1.47-9.12)	0.005†

Notes: †P<0.05, statistically significant.
